# Sorafenib and CuB exert synergistic antitumor effects against hepatocellular carcinoma cells via inhibition of STAT3 phosphorylation

**DOI:** 10.1002/2211-5463.13035

**Published:** 2020-11-27

**Authors:** Xiaoli Wang, Hua Li, Dong Li, Yudi Bai, Yao Zhang, Xue Yan, Jin Li, Ri Zhao, Jiahui Liu, Wei Liu, Maolin Shi, Cheng Xu, Tai Yang, Tao Zhang

**Affiliations:** ^1^ Department of Cancer Hospital of Chengdu University of Traditional Chinese Medicine Chengdu China; ^2^ Clinical School of Medicine Chengdu University of Traditional Chinese Medicine Chengdu China; ^3^ Cancer Center The General Hospital of Western Theater Command Chengdu China; ^4^ Basic School of Medicine Southwest Jiaotong University Chengdu China; ^5^ College of Pharmacy Chengdu Medical College Chengdu China; ^6^ Scientific Research Center Chengdu Medical College Chengdu China; ^7^ Basic School of Medicine Chengdu University of Traditional Chinese Medicine Chengdu China; ^8^ Clinical School of Medicine Southwest Medical University Luzhou China

**Keywords:** CuB, EGF, hepatocellular carcinoma, Sorafenib, STAT3, synergism

## Abstract

Sorafenib, the first‐line agent for treatment of advanced hepatocellular carcinoma (HCC), improves median overall survival by approximately 3 months. In the present study, we investigated whether sorafenib combined with cucurbitacin B (CuB), a natural tetracyclic triterpenoid isolated from *Cucurbitaceae*, exerts enhanced antitumor effects against HCC. Cell viability and colony formation ability were detected by cell‐counting kit‐8 and colony formation assays. Cell cycle and apoptosis were analyzed by flow cytometry. Protein expression was detected by western blotting. HepG2 xenografts in nude mice were used to evaluate *in vivo* antitumor effects. We report that sorafenib and CuB exhibited synergistic effects on cellular proliferation inhibition and cell apoptosis induction, but not on cell cycle arrest. Furthermore, combination treatment enhanced levels of cleaved caspase 3 and cleaved caspase 9, but suppressed phosphorylation of STAT3. Epidermal growth factor, a potent stimulator of signal transducer and activator of transcription‐3 (STAT3), promoted cell viability and colony formation ability, whereas combination treatment exerted inhibitory effects on epidermal growth factor‐induced STAT3 phosphorylation. Finally, HepG2 xenograft mice cotreated with sorafenib and CuB exhibited reduced tumor progression without notable weight loss. In conclusion, sorafenib and CuB exert synergistic antitumor effects through a pathway that may involve STAT3 phosphorylation, and this may represent a promising therapeutic approach for treatment of HCC.

## Graphical abstract

The present study reports that the combined use of sorafenib and cucurbitacin B can inhibit cell proliferation and induce cell apoptosis *in vitro* at the same time as reducing tumor progression *in vivo*, demonstrating synergistic antitumor effects against hepatocellular carcinoma cells. In addition, combination treatment inhibited epidermal growth factor‐induced signal transducer and activator of transcription‐3 (STAT3) phosphorylation. These findings suggest that inhibition of STAT3 phosphorylation may be involved in the synergistic molecular mechanisms.

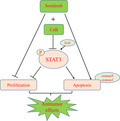

**Abbreviations**
CCK‐8: cell counting kit‐8
CI: combination index
CuB: cucurbitacin B
EGF: epidermal growth factor
FITC: fluorescein 5‐isothiocyanate
HCC: hepatocellular carcinoma
IC_50_: half maximal inhibitory concentration
PI: propidium iodide
STAT3: signal transducer and activator of transcription‐3

## Introduction

Hepatocellular carcinoma is the fifth most commonly diagnosed cancer and the third leading cause of cancer‐related death worldwide [1]. The common causes of hepatocellular carcinoma (HCC) are attributed to chronic infection by hepatitis B or C virus infection, alcohol abuse, metabolic syndrome and non‐alcoholic fatty liver diseases [2, 3]. Currently, the majority of patients are diagnosed in an advanced stage, which means that conventional therapies such as surgical resection, radiofrequency, ablation, transarterial chemoembolization and liver transplantation are no longer eligible for these patients [4, 5]. Sorafenib is the first multikinase inhibitor approved by the Food and Drug Administration for treatment of advanced HCC in 2007 by targeting the RAF/MEK/ERK pathway and tyrosine kinases vascular endothelial growth factor receptor/platelet‐derived growth factor receptor [6, 7]. Previous reports have established that sorafenib could improve median overall survival by approximately 3 months when used as a first‐line treatment [6, 8]. Despite an initial response, most patients develop disease progression as a result of sorafenib resistance and serious side effects (hand‐foot skin reaction, diarrhea, hypertension, abdominal pain, weight loss, etc.) [9]. Therefore, it is imperative to investigate other effective agents to combine with sorafenib with the aim of improving antitumor therapeutic effects and suppressing disease progression in the management of hepatocellular carcinoma.

Cucurbitacin B (CuB) belongs to a group of tetracyclic triterpenoids [10] that have been isolated from the plant family of *Cucurbitaceae* [11]. Medical plants rich in CuB have long been used in traditional Chinese medicine to treat various diseases as a result of its wide range of pharmacological activities, such as antitumor, antimicrobial, antiviral, anti‐inflammatory, antipyretic and analgesic effects [10, 12]. For example, CuB tablets could be used for the treatment of chronic hepatitis in China [13]. It is noteworthy that the antitumor activity of CuB was widely reported in a variety of cancers, including liver cancer, lung cancer, skin cancer, colorectal cancer, breast cancer [14], laryngeal squamous cell carcinoma [15], osteosarcoma [16] and pancreatic cancer [17]. Previous studies have shown that the antitumor effects can be enhanced further when CuB is combined with curcumin in hepatoma cells [18] and also when CuB is combined with gefitinib in human colorectal cancer cell [19]. Accumulating evidence suggests that signal transducer and activator of transcription‐3 (STAT3) is a potential molecular target of CuB [20, 21]. Considering its wide antitumor effects, we hypothesized that CuB would enhance the antitumor effects of sorafenib in HCC.

Thus, the present study aimed to investigate the antitumor effects of combination treatment with sorafenib and CuB *in vitro* and *in vivo*, as well as the molecular mechanisms in HCC.

## Materials and methods

### Cell culture and reagents

Human hepatocellular carcinoma cell lines (HepG2, Huh7 and Hep3B) were obtained from the Chinese Academy of Sciences (Beijing, China), cultured in Dulbecco’s modified Eagle’s medium containing 10% fetal bovine serum and 1% penicillin/streptomycin (SV30010; HyClone, Logan, UT, USA), and incubated in 5% CO_2_ at 37 °C. Sorafenib (#S7397) was purchased from Selleckchem (Houston, TX, USA). CuB (#6199‐67‐3) was obtained from Must Biotechnology (Chengdu, China). phospho‐STAT3 (#9145S), STAT3 (#4904S), cleaved caspase 3 (#9915T), cleaved caspase 9 (#9915T), GAPDH (#2118S) and horseradish peroxidase‐conjugated secondary antibodies were purchased from Cell Signaling Technology (Beverly, MA, USA). Human epidermal growth factor (EGF) (AF‐100‐15‐100) was purchased from Peprotech (Rocky Hill, NJ, USA).

### Measurement of cell viability and evaluation of synergism

Cell viability following treatment was assessed via a cell counting kit‐8 (CCK‐8) assay. Briefly, HCC cells (5 × 10^3^ per well) were seeded in 96‐well plates and treated with the indicated concentrations of sorafenib and CuB alone for 48 h. For the combination study, we selected sorafenib (250, 500, 1000 and 2000 nm), CuB (3.125, 6.25, 12.5 and 25 nm) and combinations of them for 48 h, respectively. Then, 10 μL CCK‐8 reagent (JE603; Dojindo, Kumamoto, Japan) was added to each well and incubated for another 4 h. Absorbance at 450 nm was measured by multi‐well spectrophotometer. Half maximal inhibitory concentration (IC_50_) values were calculated using prism (GraphPad Software Inc., San Diego, CA, USA). The combined effects of two agents were evaluated via combination index (CI) values using calcusyn (Biosoft, Cambridge, UK) based on the median‐effect principle and the Chou–Talalay equation [22]. CI < 1, CI = 1 and CI > 1 represent synergistic, additive and antagonistic effects, respectively.

### Colony formation assay

The long‐term antitumor effects following treatments were evaluated by a colony formation assay. Briefly, HepG2 and Huh7 cells (1000 cells per well) were incubated overnight in six‐well plates and exposed to 2000 nm sorafenib and/or 25 nm CuB for 48 h. Next, the cells were allowed to grow for another 12 days and then were fixed using 4% paraformaldehyde and stained with crystal violet. Colonies containing more than 50 cells in each well were counted.

### Flow cytometric analysis

Cell cycle distributions were detected by flow cytometry analysis (ACEA Bioscience, San Diego, CA, USA). After treating HepG2 and Huh7 cells with sorafenib, CuB and a combination of them, respectively, for 48 h, HCC cells were fixed with ice‐cold 70% ethanol at 4 °C overnight and stained with 500 μL of propidium iodide (PI) in RNase A staining working fluid (KGA 512; Keygen, Nanjing, China) for 30 min. DNA distributions of the cells were measured by flow cytometry.

Quantification of apoptosis was also detected by flow cytometry using an annexin V‐fluorescein 5‐isothiocyanate (FITC)/PI apoptosis detection kit (KGA108; Keygen). After treatment, cells were washed with PBS and stained with annexin V/PI reagents in binding buffer for 15 min in the dark. Then, cell samples were detected by flow cytometry.

### Western blot assay

HepG2 and Huh7 cells were collected after treatment with sorafenib and/or CuB for 48 h, proteins were extracted with ice‐cold RIPA lysis buffer containing 1% phosphatase/protease inhibitor cocktail (B15001/B14001; Bimake, Houston, TX, USA) and quantitated by a bicinchoninic acid assay. Equal amounts of proteins in each group were separated by 10% SDS/PAGE, then transferred to methanol‐activated poly(vinylidene difluoride) membranes (IPVH00010; Millipore, Burlington, MA, USA). Then, membranes were blocked with 5% BSA for 1 h, probed with primary antibodies at 4 °C overnight and incubated with secondary antibodies for 1 h at room temperature. Finally, the proteins on poly(vinylidene difluoride) membranes were visualized with an Enhanced Chemiluminescence Detection Kit (WBKLS0100; Millipore) using Quantity One software (Bio‐Rad, Hercules, CA, USA). Protein expression levels were normalized with GAPDH expression.

### EGF treatment

EGF was used at a concentration of 10 ng·mL^−1^. Briefly, CCK‐8 and colony formation assays were carried out to detect the growth and colony formation abilities of HCC cells in complete medium supplemented with or without EGF for 48 h and 12 days. For the western blot assay, serum‐starved HCC cells were pretreated with or without 8000 nm sorafenib combined with 100 nm CuB for 12 h, then incubated with or without 10 ng·mL^−1^ EGF for 30 min. The remaining experimental steps were performed as described above.

### *In vivo* xenograft assay

All animal treatments were carried out in accordance with the guidelines of the Laboratory Animal Center of Chengdu Medical College (Chengdu, China). The nude mice (female BALB/c, 5 weeks old) were purchased from Jiangsu Jicui Yaokang Biotechnology (Nanjing, China) and housed under specific pathogen free conditions: 12 :12 h light/dark photocycle at 22 ± 2 °C and 60 ± 10% humidity. Standard rodent diet and water were provided *ad libitum*. After 1 week of balanced feeding, 5 × 10^6^ HepG2 cells were injected subcutaneously into the right flank of nude mice. When the subcutaneous tumors grew to an average size of 200 mm^3^, mice were randomized into four groups (*n* = 6) to receive the treatments: vehicle (0.5% sodium carboxyl methylcellulose), sorafenib (25 mg·kg^−1^) by gavage once a day, CuB (0.75 mg·kg^−1^) by intraperitoneal injection every other day, and a combination treatment of sorafenib and CuB, respectively. Tumor volumes were calculated using the formula (width)^2^ × length/2 every other day and body weight was weighed every 3 days. The mice were killed by cervical dislocation on day 21 and tumors were excised, photographed and weighed.

### Statistical analysis

All results were obtained in triplicate from at least three independent experiments. Data are presented as the mean ± SD for each group. An unpaired Student’s *t*‐test was used to compare differences between two groups. *P* < 0.05 was considered statistically significant.

## Results

### The synergistic antiproliferative effects of sorafenib combined with CuB in HCC cells

**Fig. 1:**
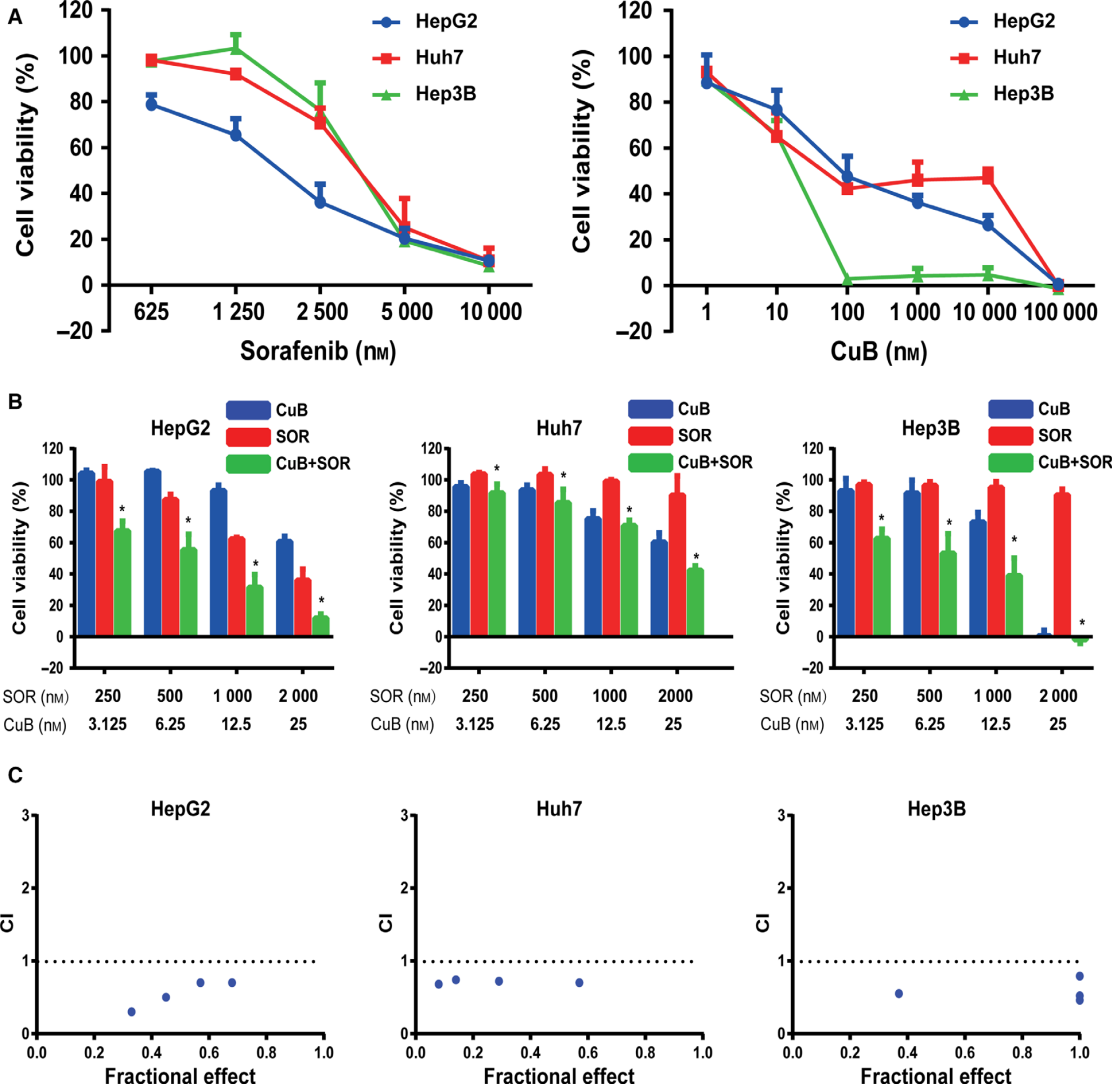
The synergistic antiproliferative effects of sorafenib and CuB in HCC cells. (A) Following treatment with the indicated concentrations of sorafenib and CuB alone for 48 h, IC_50_ values of sorafenib and CuB alone in HepG2, Huh7 and Hep3B cells were calculated using prism after the CCK‐8 assay. (B) HCC cells were treated with the indicated concentrations of sorafenib and/or CuB for 48 h. Cell viability was assessed by the CCK‐8 assay. (C) The Fa‐CI plot indicated the CI values via calcusyn. CI < 1, CI = 1 and CI > 1 represent synergistic, additive and antagonistic effects, respectively. Data are presented as the mean ± SD from three independent experiments. Differences between two groups were analyzed using Student’s *t*‐test. *P < 0.05 vs. sorafenib. SOR, sorafenib; CuB, cucurbitacin B.

To investigate the antiproliferative effects of sorafenib and/or CuB in HCC cells, HepG2, Huh7 and Hep3B cells were treated with increasing concentrations of agents, alone and in combination. Figure 1A shows that the viabilities of all tested HCC cells were inhibited in a concentration‐dependent manner following treatment with the indicated concentrations of sorafenib or CuB alone for 48 h. The IC_50_ value of sorafenib was estimated to be 1792 nm (HepG2), 3431 nm (Huh7) and 3447 nm (Hep3B), and that of CuB was 57 nm (HepG2), 47 nm (Huh7) and 15 nm (Hep3B). The growth‐suppressive effects of sorafenib were significantly enhanced when combined with CuB at a concentration ratio of 80 : 1 (Fig. 1B). In addition, as shown in Fig. 1C, CI analysis showed that combination treatment had synergistic effects in three HCC cell lines (CI < 1). Taken together, the results indicated that sorafenib or CuB alone exerted antiproliferative effects, whereas a combination demonstrated synergistic killing effects in HCC cells.

### Combination of sorafenib and CuB synergistically inhibits colony formation in HCC cells

**Fig. 2:**
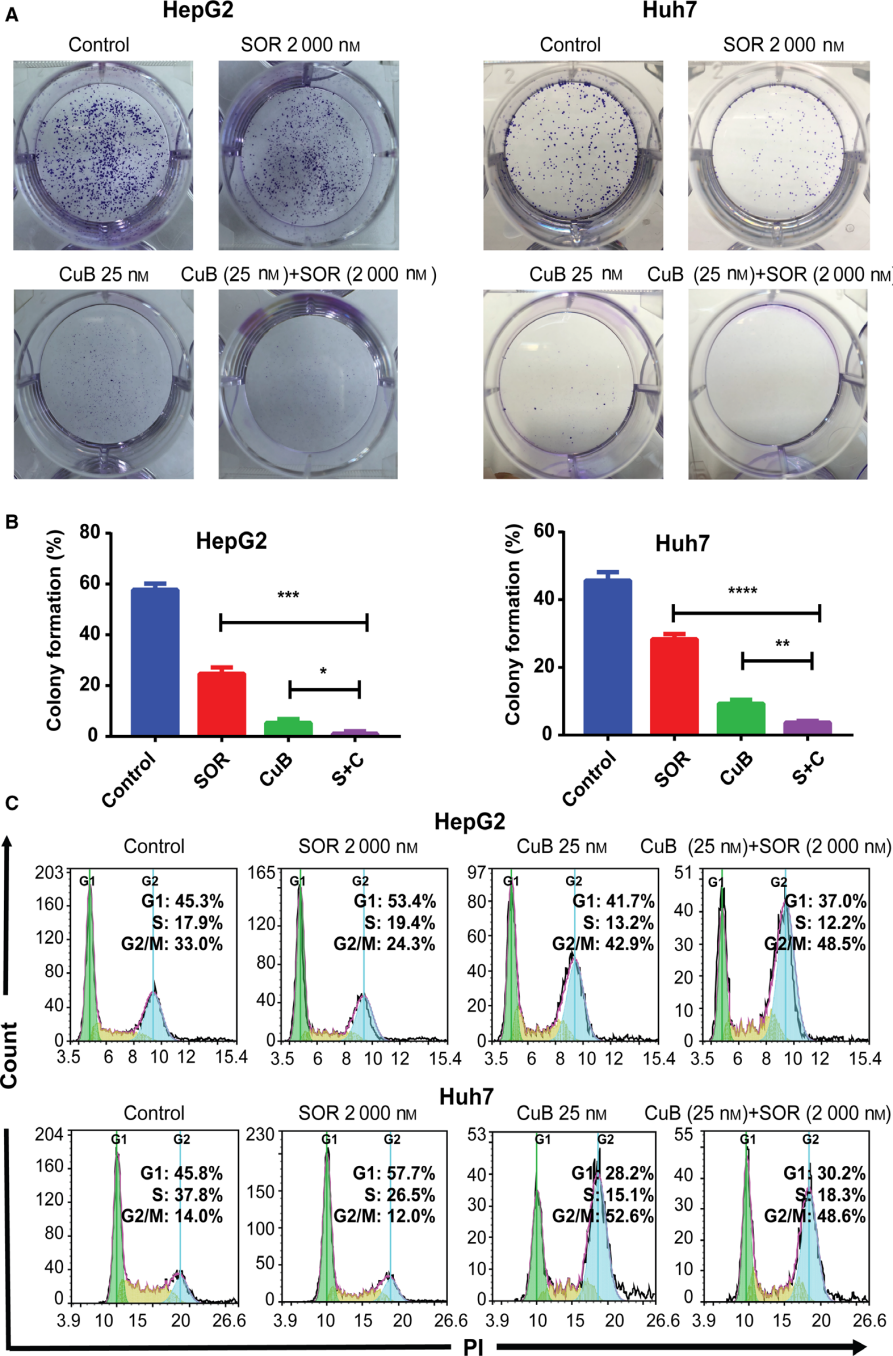
Combination of sorafenib and CuB decreases colony formation synergistically and arrests the cell cycle at the G2/M phase in HCC cells. (A, B) Following treatment with 2000 nm sorafenib and/or 25 nm CuB for 48 h, the number of colonies in HepG2 and Huh7 cells was recorded. (C) The DNA content distributions of the cell cycle were detected by flow cytometry with PI staining after treatment for 48 h. Data are presented as the mean ± SD from three independent experiments. Differences between two groups were analyzed using Student’s *t*‐test. *P < 0.05, **P < 0.01, ***P < 0.001 and ****P < 0.0001 vs. the corresponding group.

The long‐term antitumor potential of sorafenib and/or CuB was further evaluated by a colony formation assay. As shown in Fig. 2A,B, sorafenib or CuB treatment alone inhibited colony formation to some extent, whereas combination treatment of sorafenib and CuB dramatically decreased the number of colonies in HepG2 and Huh7 cells. These results indicated that a combination of sorafenib and CuB exhibited synergistic long‐term antiproliferative effects in HCC cells.

### The cell cycle is arrested at different phases by sorafenib and/or CuB in HCC cells

To determine whether combination treatment could arrest the cell cycle, a PI staining assay with flow cytometry was utilized to detect the DNA content distributions of each group. The results shown in Fig. 2C indicated that treatment with sorafenib alone lead to an increase in the G1 phase (53.4% in HepG2 and 57.7% in Huh7), whereas treatment with CuB alone resulted in an increase in the G2/M phase (42.9% in HepG2 and 52.6% in Huh7). However, treatment with a combination together resulted in an increase in the G2/M phase, the post stage of DNA synthesis (48.5% in HepG2 and 48.6% in Huh7). These results suggested that a combination of sorafenib and CuB could induce G2/M cell cycle arrest in HCC cells, although not in a synergistic manner.

### Combination of sorafenib and CuB synergistically induces caspase‐dependent apoptosis in HCC cells

**Fig. 3:**
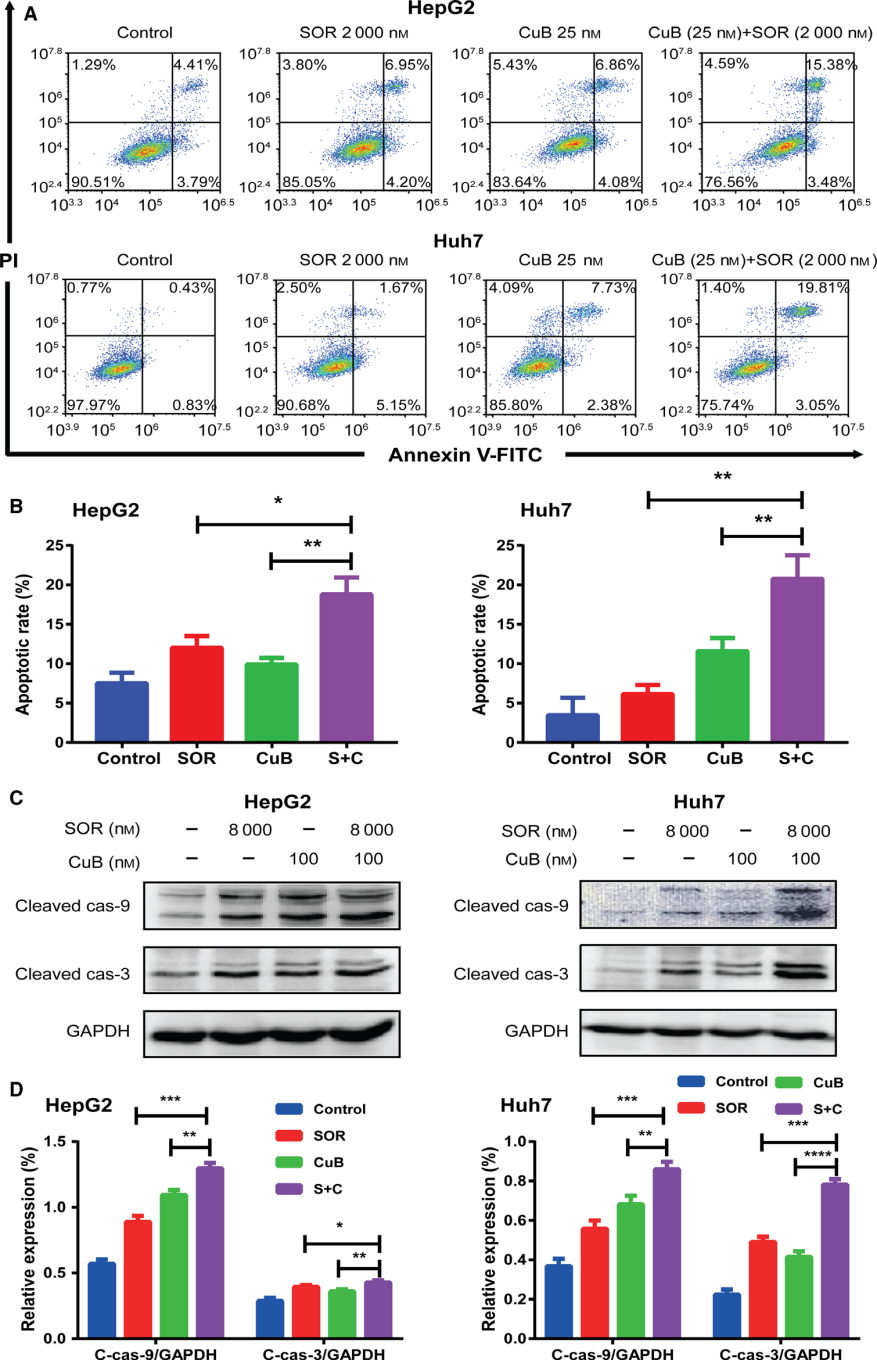
Combination of sorafenib and CuB synergistically induces caspase‐dependent apoptosis in HCC cells. (A, B) HepG2 and Huh7 cells were treated with 2000 nm sorafenib and/or 25 nm CuB for 48 h, An annexin V‐FITC/PI staining assay with flow cytometry was used to detect the apoptotic rates. (C, D) The expression levels of cleaved caspase 3 and cleaved caspase 9 were detected by western blotting after treatment for 48 h. GAPDH was used as a loading control. Data are presented as the mean ± SD from three independent experiments. Differences between two groups were analyzed using Student’s *t*‐test. *P < 0.05, **P < 0.01, ***P < 0.001 and ****P < 0.0001 vs. the corresponding group.

To investigate whether combination treatment could induce cell apoptosis, the percentage of annexin V‐FITC/PI‐positive cells was measured by flow cytometry. As shown in Fig. 3A,B, the apoptotic rate in sorafenib and CuB treated HepG2 cells was 12.0 ± 1.5% and 9.9 ± 0.8%, respectively, whereas it increased to 18.8 ± 2.2% following combination treatment. Similar to the results for the HepG2 cells, the flow cytometric analysis in Huh7 cells indicated that the apoptotic rate of sorafenib and CuB alone treatment was 6.2 ± 1.2% and 11.6 ± 1.7%, respectively, whereas that of combination treatment was 20.8 ± 3.0%. Moreover, the results for apoptosis‐related protein expression obtained by western blotting, as shown in Fig. 3C,D, showed that combination treatment lead to an increased accumulation of cleaved caspase 3 and cleaved caspase 9 in HepG2 and Huh7 cells compared to monotreatment. These results revealed that combined sorafenib with CuB treatment synergistically induced caspase‐dependent cell apoptosis in HCC cells.

### Inhibition of STAT3 phosphorylation is involved in the synergistic antitumor effects of combination treatment in HCC cells

**Fig. 4:**
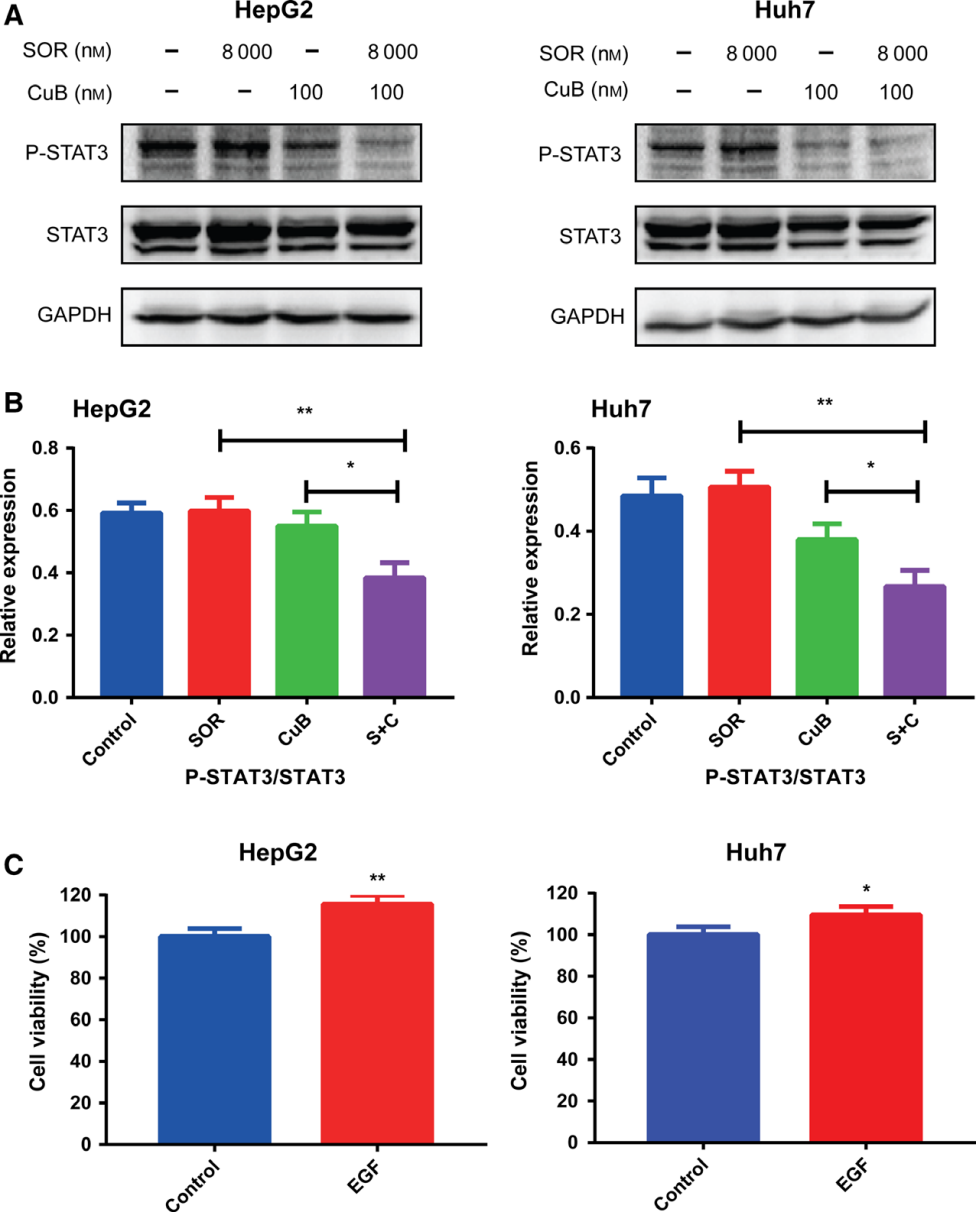
Inhibition of STAT3 phosphorylation is involved in the synergistic antitumor effects of combination treatment and EGF promotes cell growth in HCC cells. (A, B) The expression levels of phospho‐STAT3 and STAT3 were analyzed by western blotting following 48 h of treatment with 8000 nm sorafenib and/or 100 nm CuB. GAPDH was used as a loading control. (C) The cell viability was detected with or without EGF for 48 h. Data are presented as the mean ± SD from three independent experiments. Differences between two groups were analyzed using Student’s *t*‐test. *P < 0.05 and **P < 0.01 vs. the corresponding group.

**Fig. 5:**
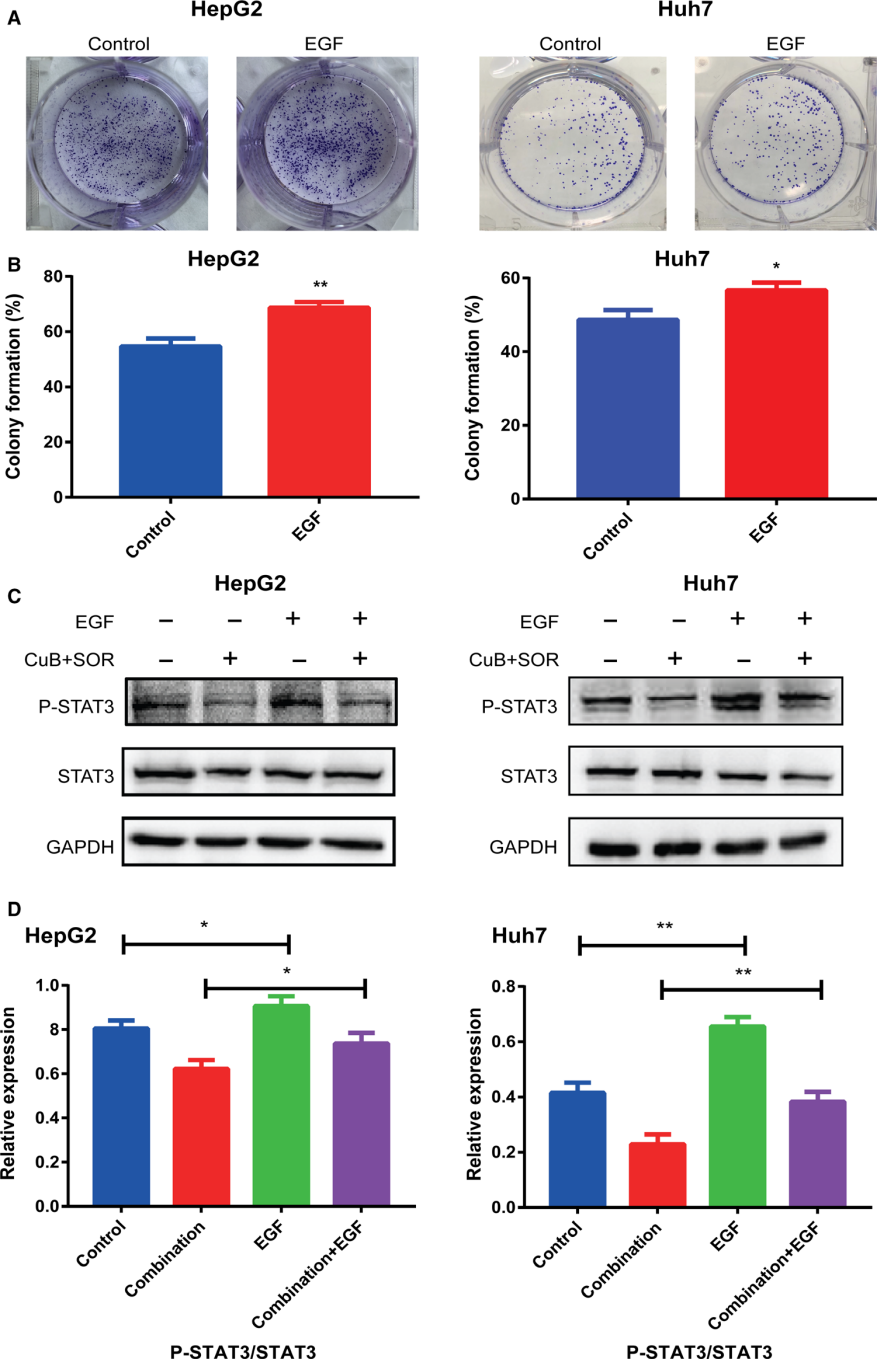
EGF promotes colony formation and combination treatment exerts inhibitory effects on EGF activated‐STAT3 phosphorylation. (A, B) Colony formation was detected with or without EGF for 12 days. (C, D) Serum‐starved HCC cells were pretreated with or without 8000 nm sorafenib combined with 100 nm CuB for 12 h, then incubated with or without 10 ng·mL^−1^ EGF for 30 min. The expression levels of phospho‐STAT3 and STAT3 were analyzed by western blotting. GAPDH was used as a loading control. Data are presented as the mean ± SD from three independent experiments. Differences between two groups were analyzed using Student’s *t*‐test. *P < 0.05 and **P < 0.01 vs. the corresponding group.

To explore the underlying molecular mechanisms driving the synergistic antitumor effects, we detected the expression levels of drug‐treated HCC cell STAT3 phosphorylation, which was reported to be aberrantly activated in HCC [23, 24]. Western blotting, as shown in Fig. 4A,B, indicated that the expression levels of total STAT3 remained almost unchanged in each group. Compared to control, sorafenib had no inhibitory effect on STAT3 phosphorylation, whereas CuB significantly downregulated STAT3 phosphorylation and suppressed it more significantly when combined with sorafenib, implying that inhibition of STAT3 phosphorylation could be involved in the sorafenib/CuB‐mediated molecular mechanisms. In addition, we used EGF, a potent stimulator of the STAT3 pathway [25] to investigate whether the antitumor effects of combination treatment are dependent of STAT3 phosphorylation. As shown in Figs 4C and 5A,B, cell viabilities and colony formation abilities were enhanced by EGF in HepG2 and Huh7 cells. More importantly, western blotting indicated that STAT3 phosphorylation was activated by EGF (Fig. 5C,D), whereas it was inhibited by CuB and combination treatment (Figs 4A and 5C). Furthermore, we also found that combination treatment groups still exerted inhibitory effects on EGF activated‐STAT3 phosphorylation. These findings demonstrated that cell growth and STAT3 phosphorylation were positively associated with EGF, and that inhibition of STAT3 phosphorylation at least partly participated in the synergistic molecular mechanisms.

### The synergistic antitumor effects of sorafenib and CuB in the HepG2 xenograft model

**Fig. 6:**
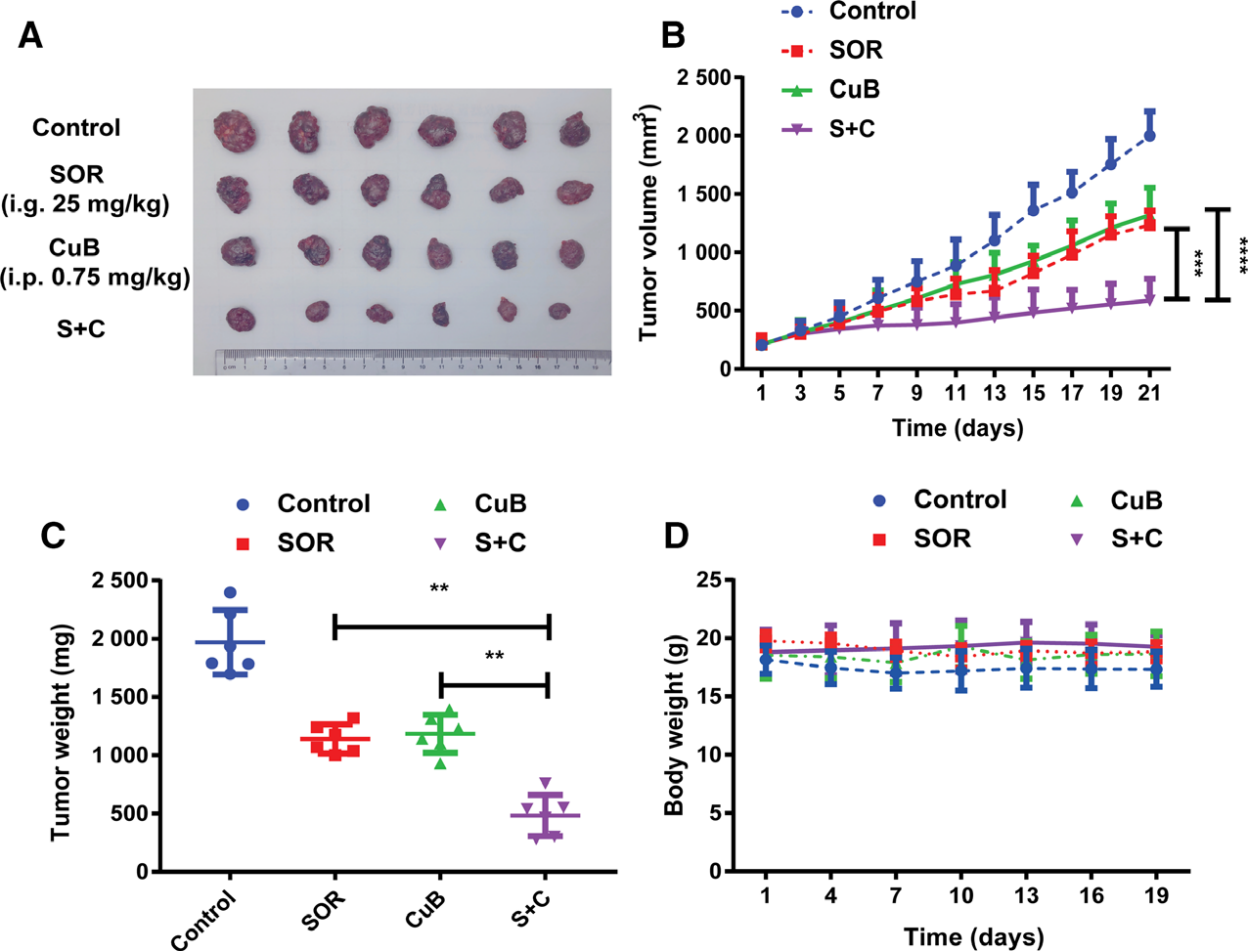
The synergistic antitumor effect of sorafenib and CuB in the HepG2 xenograft model. Following treatment with sorafenib (25 mg·kg^−1^) by gavage once a day and/or CuB (0.75 mg·kg^−1^) by intraperitoneal injection every other day, nude mice were killed on day 21 and tumors were excised. (A, B) Tumor volumes, (C) tumor weights and (D) body weights of the tumor‐bearing mice in each group. Data are presented as the mean ± SD from three independent experiments. Differences between two groups were analyzed using Student’s *t*‐test. **P < 0.01, ***P < 0.001 and ****P < 0.0001 vs. the corresponding group.

To further confirm whether combination treatment could have synergistic antitumor effect *in vivo*, we established human HCC xenografts in nude mice. As shown in Fig. 6A,B, tumor growth was moderately inhibited by monotherapy alone, whereas it was strongly inhibited by a combination. Consistent with the results for tumor volumes, the average tumor weight with respect to control, sorafenib, CuB and the combination group was 1969 ± 277, 1141 ± 126, 1185 ± 164, 484 ± 177 mg (Fig. 6C), respectively, with a significant statistical difference between groups (*P* < 0.01). No losses in body weights were observed in any of the groups, indicating that combination treatment had no obvious side effects *in vivo* (Fig. 6D). Therefore, the results demonstrated that combination treatment also had synergistic antitumor effects *in vivo*, implying that a combination of sorafenib and CuB may have superior antitumor efficacy compared to monotherapy in the clinic.

## Discussion

Sorafenib has been used as a first‐line antitumor targeted drug with respect to monotherapy for unresectable or metastatic HCC over the past 10 years, although it can only prolong the survival time of advanced HCC patients by approximately 3 months [6]. Moreover, the use of high doses, serious adverse effects and drug resistance lead to low compliance and treatment interruptions. Therefore, it is important to explore new therapeutic strategies for coping with these problems. CuB is a natural compound that has a wide spectrum of potent antitumor activities against many types of cancers. Additionally, a previous study reported that CuB may have therapeutic value in suppressing the growth in human HCC cells [26], and another study indicated that CuB could induce apoptosis and cell cycle arrest in BEL‐7402 human HCC [27], rendering CuB as an ideal supplementary to HCC. However, whether combining sorafenib with CuB could improve the efficacy of sorafenib remained be explored. In the present study, we demonstrated for the first time that a combination of sorafenib and CuB demonstrated synergistic antitumor effects in HCC cells *in vitro* and *in vivo*.

We found that sorafenib could synergistically increase cytotoxic effects and decrease colony formation abilities against HCC cells when combined with CuB. The CI values obtained using calcusyn software further confirmed the synergistic antitumor effects. The possible reasons contributing to the synergistic antitumor effects were investigated. The results obtained showed that the cell cycle was arrested at the G1 and G2/M phase by sorafenib and CuB alone, whereas it was arrested at the G2/M phase by a combination treatment in HCC cells, indicating that cell cycle arrest may not be responsible for the synergistic antitumor effects. As expected, the results of flow cytometry indicated that combination treatment induced cell apoptosis to a significantly greater extent compared to either agent alone, suggesting that cell apoptosis induced by combination treatment may account for the synergistic antitumor effects in HCC cells.

It is well known that cell apoptosis plays an important role in normal cell growth and cancer development. The mitochondrial apoptotic pathway has long been considered as a key pathway for cell apoptosis [28]. Caspase 9 and caspase 3 are representative downstream executive proteins that lead to cell apoptosis [29]. In the present study, we found that both sorafenib and CuB alone lead to cleaved caspase 3 and cleaved caspase 9 accumulation, similar to that reported previously in HCC cells for sorafenib [30] or CuB alone [18]. Compared to monotherapy, combination treatment markedly upregulated the expression levels of cleaved caspase 3 and cleaved caspase 9, suggesting that sorafenib combined with CuB treatment synergistically induced caspase‐dependent cell apoptosis in HCC cells.

Furthermore, a previous study reported that STAT3 dephosphorylation could take part in CuB inhibitory growth [26], whereas STAT3 phosphorylation also induced resistance to sorafenib in HCC cells [31]. Hence, we mainly focused on STAT3, a well‐known aberrantly activated oncogene in many human cancers, which relays cytokine receptor‐generated signals into the nucleus to stimulate cell proliferation, prevent apoptosis, promote angiogenesis and facilitate tumor immune evasion [32, 33]. As shown in Fig. 4A,B, western blotting showed that sorafenib did not influence STAT3 phosphorylation, consistent with a study reporting sorafenib had little impact or even slightly increased phosphorylated STAT3 [34]; however, we found that STAT3 phosphorylation was inhibited moderately by CuB and significantly inhibited by combination treatment. The results of western blotting showed that STAT3 phosphorylation was a potential target, which prompted us to further validate its role in synergistic antitumor effects. The accumulating literature indicates that some types of tumors produce EGF in excess, and EGF could activate some signal transduction pathways such as the phosphoinositide 3‐kinase/AKT, RAS/ERK and JAK/STAT pathways, by binding to cell surface receptors [35]. Li *et al*. [36] confirmed that overexpression of EGF receptor could enhance the phosphorylation of STAT3, and EGFR‐SRC‐STAT3 signaling results in resistance to sorafenib in HCC. To further understand the role of EGF with respect to proliferation and the sorafenib/CuB‐mediated molecular mechanism in HCC, as shown in Figs 4C and 5A,B, EGF‐enhanced cell viabilities and colony formation abilities were identified, similar to a previous study suggesting that the EGF receptor showed a correlation with proliferating activity in HCC [37]. Moreover, consistent the result by Guren *et al*. [38] reporting that EGF could activate several STAT proteins in liver, the present study showed that STAT3 phosphorylation was enhanced by EGF (Fig. 5C,D). It is worth noting that combination treatment groups still exerted inhibitory effects on EGF activated‐STAT3 phosphorylation. These experimental findings indicated that the synergistic antitumor effect of sorafenib in combination with CuB is at least partly mediated by STAT3 phosphorylation. However, the sorafenib/CuB‐mediated molecular mechanisms might be interactive and complicated, and this needs to be explored further for other molecules in future studies. Finally, the HepG2 xenograft assay showed the mice cotreated with sorafenib and CuB had reduced tumor progression without notable weight loss, not only further demonstrating synergistic antitumor effects *in vivo*, but also indicating combination treatment is a safe and effective therapeutic approach for HCC.

In conclusion, the present study has revealed that a combination of sorafenib and CuB had synergistic antitumor effects on cellular proliferation and apoptosis in HCC cells, and also that inhibition of STAT3 phosphorylation was involved in the synergistic molecular mechanisms. Our findings suggest that a combination of sorafenib and CuB may represent a novel and safe therapeutic approach for the management of HCC.

## Conflict of interests

The authors declare that they have no conflicts of interest.

## Author contributions

WXL, LH, LD and YT conceived and supervised the study. ZT provided funding. WXL, LH, LD and YT designed the experiments. BYD, LW, and SML provided tools and reagents. WXL participated in performing all of the assays. BYD, LJ and YX performed western blot and animal assays. XC and LJH performed CCK‐8, colony formation and western blot assays. LH analyzed data from the CCK‐8 and colony formation assays. ZR performed flow cytometric analysis. LD and ZT analyzed data from western blot and animal assays. WXL and YT drew the graphs and prepared the figures. WXL wrote the manuscript. WXL, LH, YT and ZT made revisions to the manuscript.

## Data Availability

The datasets analyzed during the present study are available from the corresponding author upon reasonable request.

## References

[1] Siegel RL and Miller KD (2019) Cancer statistics, 2019. CA Cancer J Clin 69, 7–34. 30620402 10.3322/caac.21551

[2] Chan SL , Wong VW , Qin S and Chan HL (2016) Infection and cancer: the case of hepatitis B. J Clin Oncol 34, 83–90. 26578611 10.1200/JCO.2015.61.5724

[3] Gao Q , Zhu H , Dong L , Shi W , Chen R , Song Z , Huang C , Li J , Dong X , Zhou Y *et al* (2019) Integrated proteogenomic characterization of HBV‐related hepatocellular carcinoma. Cell 179, 561–577.e22. 31585088 10.1016/j.cell.2019.08.052

[4] Yarchoan M , Agarwal P , Villanueva A , Rao S , Dawson LA , Llovet JM , Finn RS and Groopman JD (2019) Recent developments and therapeutic strategies against hepatocellular carcinoma. Cancer Res 79, 4326–4330. 31481419 10.1158/0008-5472.CAN-19-0803 PMC8330805

[5] Villanueva A (2019) Hepatocellular carcinoma. N Engl J Med 380, 1450–1462. 30970190 10.1056/NEJMra1713263

[6] Llovet JM , Ricci S , Mazzaferro V , Hilgard P , Gane E , Blanc JF , de Oliveira AC , Santoro A , Raoul JL , Forner A *et al* (2008) Sorafenib in advanced hepatocellular carcinoma. N Engl J Med 359, 378–390. 18650514 10.1056/NEJMoa0708857

[7] Wilhelm SM , Adnane L , Newell P , Villanueva A , Llovet JM and Lynch M (2008) Preclinical overview of sorafenib, a multikinase inhibitor that targets both Raf and VEGF and PDGF receptor tyrosine kinase signaling. Mol Cancer Ther 7, 3129–3140. 18852116 10.1158/1535-7163.MCT-08-0013 PMC12261297

[8] Cheng AL , Kang YK , Chen Z , Tsao CJ , Qin S , Kim JS , Luo R , Feng J , Ye S , Yang TS *et al* (2009) Efficacy and safety of sorafenib in patients in the Asia‐Pacific region with advanced hepatocellular carcinoma: a phase III randomised, double‐blind, placebo‐controlled trial. Lancet Oncol 10, 25–34. 19095497 10.1016/S1470-2045(08)70285-7

[9] Zhu YJ , Zheng B , Wang HY and Chen L (2017) New knowledge of the mechanisms of sorafenib resistance in liver cancer. Acta Pharmacol Sin 38, 614–622. 28344323 10.1038/aps.2017.5 PMC5457690

[10] Hussain H , Green IR , Saleem M , Khattak KF , Irshad M and Ali M (2019) Cucurbitacins as anticancer agents: a patent review. Recent Pat Anticancer Drug Discov 14, 133–143. 30451116 10.2174/1574892813666181119123035

[11] Chen JC , Chiu MH , Nie RL , Cordell GA and Qiu SX (2005) Cucurbitacins and cucurbitane glycosides: structures and biological activities. Nat Prod Rep 22, 386–399. 16010347 10.1039/b418841c

[12] Luo WW , Zhao WW , Lu JJ , Wang YT and Chen XP (2018) Cucurbitacin B suppresses metastasis mediated by reactive oxygen species (ROS) via focal adhesion kinase (FAK) in breast cancer MDA‐MB‐231 cells. Chin J Nat Med 16, 10–19. 29425586 10.1016/S1875-5364(18)30025-6

[13] Wang Z , Zhu W , Gao M , Wu C , Yang C , Yang J , Wu G , Yang B and Kuang H (2017) Simultaneous determination of cucurbitacin B and cucurbitacin E in rat plasma by UHPLC‐MS/MS: a pharmacokinetics study after oral administration of cucurbitacin tablets. J Chromatogr B Analyt Technol Biomed Life Sci 1065–1066, 63–69. 10.1016/j.jchromb.2017.09.024 28946127

[14] Dandawate P and Subramaniam D (2020) Cucurbitacin B and I inhibits colon cancer growth by targeting the Notch signaling pathway. Sci Rep 10, 1290. 31992775 10.1038/s41598-020-57940-9 PMC6987129

[15] Liu T , Peng H , Zhang M , Deng Y and Wu Z (2010) Cucurbitacin B, a small molecule inhibitor of the Stat3 signaling pathway, enhances the chemosensitivity of laryngeal squamous cell carcinoma cells to cisplatin. Eur J Pharmacol 641, 15–22. 20483353 10.1016/j.ejphar.2010.04.062

[16] Zhang ZR , Gao MX and Yang K (2017) Cucurbitacin B inhibits cell proliferation and induces apoptosis in human osteosarcoma cells via modulation of the JAK2/STAT3 and MAPK pathways. Exp Ther Med 14, 805–812. 28673003 10.3892/etm.2017.4547 PMC5488743

[17] Zhou J , Zhao T , Ma L , Liang M , Guo YJ and Zhao LM (2017) Cucurbitacin B and SCH772984 exhibit synergistic anti‐pancreatic cancer activities by suppressing EGFR, PI3K/Akt/mTOR, STAT3 and ERK signaling. Oncotarget 8, 103167–103181. 29262554 10.18632/oncotarget.21704 PMC5732720

[18] Sun Y , Zhang J , Zhou J , Huang Z , Hu H , Qiao M , Zhao X and Chen D (2015) Synergistic effect of cucurbitacin B in combination with curcumin via enhancing apoptosis induction and reversing multidrug resistance in human hepatoma cells. Eur J Pharmacol 768, 28–40. 26452516 10.1016/j.ejphar.2015.10.003

[19] Yar Saglam AS , Alp E , Elmazoglu Z and Menevse S (2016) Treatment with cucurbitacin B alone and in combination with gefitinib induces cell cycle inhibition and apoptosis via EGFR and JAK/STAT pathway in human colorectal cancer cell lines. Hum Exp Toxicol 35, 526–543. 26183715 10.1177/0960327115595686

[20] Zhang M , Bian ZG , Zhang Y , Wang JH , Kan L , Wang X , Niu HY and He P (2014) Cucurbitacin B inhibits proliferation and induces apoptosis via STAT3 pathway inhibition in A549 lung cancer cells. Mol Med Rep 10, 2905–2911. 25242136 10.3892/mmr.2014.2581 PMC4227420

[21] Xu J , Chen Y , Yang R , Zhou T , Ke W , Si Y , Yang S , Zhang T , Liu X , Zhang L *et al* (2020) Cucurbitacin B inhibits gastric cancer progression by suppressing STAT3 activity. Arch Biochem Biophys 684, 108314. 32088220 10.1016/j.abb.2020.108314

[22] Chou TC (2006) Theoretical basis, experimental design, and computerized simulation of synergism and antagonism in drug combination studies. Pharmacol Rev 58, 621–681. 16968952 10.1124/pr.58.3.10

[23] Gu FM , Li QL , Gao Q , Jiang JH , Huang XY , Pan JF , Fan J and Zhou J (2011) Sorafenib inhibits growth and metastasis of hepatocellular carcinoma by blocking STAT3. World J Gastroenterol 17, 3922–3932. 22025881 10.3748/wjg.v17.i34.3922 PMC3198022

[24] Xie L , Zeng Y , Dai Z , He W , Ke H , Lin Q , Chen Y , Bu J , Lin D and Zheng M (2018) Chemical and genetic inhibition of STAT3 sensitizes hepatocellular carcinoma cells to sorafenib induced cell death. Int J Biol Sci 14, 577–585. 29805309 10.7150/ijbs.22220 PMC5968850

[25] Zhao FL and Qin CF (2019) EGF promotes HIF‐1α expression in colorectal cancer cells and tumor metastasis by regulating phosphorylation of STAT3. Eur Rev Med Pharmacol Sci 23, 1055–1062. 30779072 10.26355/eurrev_201902_16993

[26] Zhang M , Zhang H , Sun C , Shan X , Yang X , Li‐Ling J and Deng Y (2009) Targeted constitutive activation of signal transducer and activator of transcription 3 in human hepatocellular carcinoma cells by cucurbitacin B. Cancer Chemother Pharmacol 63, 635–642. 18521604 10.1007/s00280-008-0780-0

[27] Chan KT , Meng FY , Li Q , Ho CY , Lam TS , To Y , Lee WH , Li M , Chu KH and Toh M (2010) Cucurbitacin B induces apoptosis and S phase cell cycle arrest in BEL‐7402 human hepatocellular carcinoma cells and is effective via oral administration. Cancer Lett 294, 118–124. 20153103 10.1016/j.canlet.2010.01.029

[28] Bock FJ and Tait SWG (2020) Mitochondria as multifaceted regulators of cell death. Nat Rev Mol Cell Biol 21, 85–100. 31636403 10.1038/s41580-019-0173-8

[29] Inoue S , Browne G , Melino G and Cohen GM (2009) Ordering of caspases in cells undergoing apoptosis by the intrinsic pathway. Cell Death Differ 16, 1053–1061. 19325570 10.1038/cdd.2009.29

[30] Jiang C , Xu R , Li XX , Zhou YF , Xu XY , Yang Y , Wang HY and Zheng XFS (2018) Sorafenib and carfilzomib synergistically inhibit the proliferation, survival, and metastasis of hepatocellular carcinoma. Mol Cancer Ther 17, 2610–2621. 30224431 10.1158/1535-7163.MCT-17-0541 PMC9110113

[31] Su JC , Tseng PH , Wu SH , Hsu CY , Tai WT , Li YS , Chen IT , Liu CY , Chen KF and Shiau CW (2014) SC‐2001 overcomes STAT3‐mediated sorafenib resistance through RFX‐1/SHP‐1 activation in hepatocellular carcinoma. Neoplasia 16, 595–605. 25047655 10.1016/j.neo.2014.06.005 PMC4198826

[32] Zhao M , Jiang B and Gao FH (2011) Small molecule inhibitors of STAT3 for cancer therapy. Curr Med Chem 18, 4012–4018. 21824090 10.2174/092986711796957284

[33] Guanizo AC , Fernando CD , Garama DJ and Gough DJ (2018) STAT3: a multifaceted oncoprotein. Growth Factors 36, 1–14. 29873274 10.1080/08977194.2018.1473393

[34] Xu J , Lin H , Li G , Sun Y , Shi L , Ma WL , Chen J , Cai X and Chang C (2017) Sorafenib with ASC‐J9(®) synergistically suppresses the HCC progression via altering the pSTAT3‐CCL2/Bcl2 signals. Int J Cancer 140, 705–717. 27668844 10.1002/ijc.30446 PMC5215679

[35] Henson ES and Gibson SB (2006) Surviving cell death through epidermal growth factor (EGF) signal transduction pathways: implications for cancer therapy. Cell Signal 18, 2089–2097. 16815674 10.1016/j.cellsig.2006.05.015

[36] Li R , Yanjiao G , Wubin H , Yue W , Jianhua H , Huachuan Z , Rongjian S and Zhidong L (2017) Secreted GRP78 activates EGFR‐SRC‐STAT3 signaling and confers the resistance to sorafeinib in HCC cells. Oncotarget 8, 19354–19364. 28423613 10.18632/oncotarget.15223 PMC5386689

[37] Ito Y , Takeda T , Sakon M , Tsujimoto M , Higashiyama S , Noda K , Miyoshi E , Monden M and Matsuura N (2001) Expression and clinical significance of erb‐B receptor family in hepatocellular carcinoma. Br J Cancer 84, 1377–1383. 11355950 10.1054/bjoc.2000.1580 PMC2363640

[38] Guren TK , Abrahamsen H , Thoresen GH , Babaie E , Berg T and Christoffersen T (1999) EGF‐induced activation of Stat1, Stat3, and Stat5b is unrelated to the stimulation of DNA synthesis in cultured hepatocytes. Biochem Biophys Res Commun 258, 565–571. 10329425 10.1006/bbrc.1999.0684

